# Fabrication of a 25.2 T NMR magnet for an extreme condition user facility in China

**DOI:** 10.1093/nsr/nwae165

**Published:** 2024-05-10

**Authors:** Hui Han, Yun Shang

**Affiliations:** MRI Research Institute, Department of Radiology, Weill Medical College of Cornell University, USA; MRI Research Institute, Department of Radiology, Weill Medical College of Cornell University, USA

**Keywords:** NMR, magnet, ultra-high field, high temperature superconducting, double pancake

## Abstract

This article highlights a research study on the fabrication of a 25.2 T ultra-high field NMR magnet for an extreme condition user facility in China.

An ultra-high field magnet (>20 T) is desirable for studying the characteristics of new materials under extreme magnetic conditions [1]. Additionally, such field strength will result in resonance frequencies close to or greater than 1 GHz when applied to the study of ^1^H signals from biomolecules by nuclear magnetic resonance (NMR), which will lead to a higher signal-to-noise ratio, better sensitivity and a higher spectral resolution due to increased chemical shift dispersion [2]. However, considering the limited current density allowed by low-temperature superconducting (LTS) wires, achieving an ultra-high magnet field strength beyond 1 GHz [3] requires more advanced materials.

A number of past efforts have demonstrated that higher field strength magnets can be achieved with the use of high-temperature superconducting (HTS) materials, such as REBCO [4] and BSCCO [5] due to their higher critical magnetic fields [6]. Considering aspects of cost and reliability, a combination of LTS and HTS magnets is a reasonable choice to realize the expected high field strength [7]. A number of magnets have been fabricated for ultra-high fields using designs of an LTS magnet on the outside and HTS magnet as an insert. The LTS magnet generates the background field, while the HTS magnet can be flexibly replaced to adjust the remaining field strength.

As LTS wires have a small length-to-width ratio, they can be molded into a shape with a round or rectangular cross section, which allows magnets to be easily fabricated into solenoids through layer winding. In contrast, HTS materials are usually molded to tape structures due to their limited material properties, which indicate that the HTS wire has a quite large length-to-width ratio, making it difficult to wind solenoid coils. Although there are still some challenges in magnets constructed using a layer-winding technique, more magnets are now being wound using the double-pancake (DP) technique. The advantages of DP coils include ease of manufacture, reduced risk, and adaptability [8]. As the DP coils are independent components, variations in their fabrication would affect the overall field quality. That is one of the main engineering challenges in practice.

There are several types of high-temperature superconducting materials, and their characteristics and applications are still being explored. The REBCO and BSCCO formula have both been demonstrated to be capable of producing ultra-high field magnets, as well as having a tape shape [9]. However, the Bi-2223 (critical temperature >100 K) conductors are multifilamentary conductors whose screening currents are induced by superconducting networks made of bridged filament loops, while the REBCO conductor has a flat superconducting layer on which screening currents are freely induced [10]. The screening-current–induced magnetic fields cause time drift and field distortions that adversely affect the quality of the overall field homogeneity [11]. An inhomogeneous field may cause the magnetic resonance signal to decay faster and reduce the NMR spectrum resolution.

Although the screening current effect of the Bi-2223 tape is much weaker than that of the REBCO tape, Bi-2223 tape has greater inconsistencies when it comes to width and thickness, which makes it difficult to maintain identical dimensions of the DP coils after winding. Therefore, assembly of these DP coils directly may result in significant deviations in the magnetic field homogeneity of the central area. To overcome this challenge, the work from Wang *et al.* [12] proposed a novel random ordering method to minimize the effects of those inconsistencies in DP coils and provided a fabrication procedure for building an NMR acceptable magnet made from HTS tape of Bi-2223 at a field strength of 25.2 T (Fig. 1).

**Figure 1.**
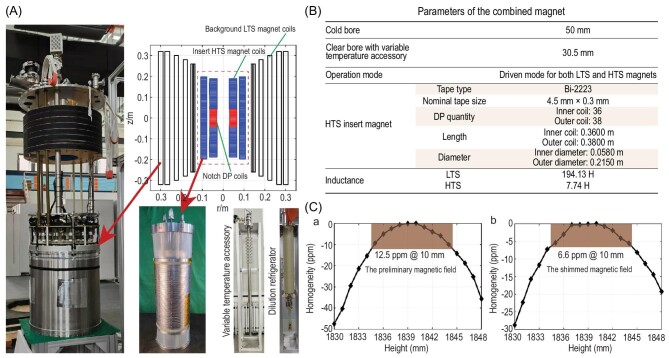
The experimental setup of the 25.2 T ultra-high field magnet, its characteristics, and the field homogeneity (adopted from Wang et al. [12]). (A) Decomposition of the experimental station for NMR with an extremely low temperature device, which enables the system to simultaneously provide a high field strength and a low temperature. (B) Dimensions of the ultra-high field magnet, work mode, and coil inductances. (C) The magnetic field homogeneity within a 10 mm sphere before and after shimming. Used with permission of IOP Publishing, Ltd, from Ref [12].

This study uses a hybrid magnet with LTS outside and HTS inside to achieve the target field. Two nested inserts were used in the HTS part, and both inserts were stacked by using DP coils. Outer DP coils were fabricated with equivalent external diameters, but with varying turns. The inner insert includes multiple DP coils and eight notch coils for field homogeneity optimization where their positions are closest to the center. To optimize the field homogeneity, they randomly re-ordered the DP coils at the outer and inner inserts multiple times and found the optimal combination. Finally, optimal winding turns were determined for each notch coil through the use of pre-winding information on the tape gauge. Winding turns of those notch coils were strictly controlled by selecting tapes with a specific thickness that closely approximated the designed values. The parameters of notch coils are presented in Table S1.

The HTS magnet inserts were fabricated using non-insulated Bi-2223 tapes and assembled using the DP technique. Upon ramping up to the field strength of 25.2 T together with the LTS magnet, the entire magnet exhibited excellent field stability, exhibiting 2.427 ppm/h on the first day and ∼0.795 ppm/h two days later, which is acceptable for NMR experiments. The measurement also demonstrated that the screening currents of the Bi-2223 magnet were significantly smaller than those of the YBCO magnet, as was expected. With the Z1 and Z2 shim coils applied, magnetic field homogeneity can be reached at 6.6 ppm over a 10 mm sphere with an acceptable level of homogeneity. The inhomogeneity of the field can be further improved in the future by the use of additional shim coils developed by the same team [13].

The results of this study demonstrated the feasibility of fabricating an ultra-high field NMR magnet using HTS Bi-2223 tapes and a DP coil technique. In addition to maintaining the advantages of the DP coil technique in terms of good stress-bearing capability, they have overcome the challenges related to inconsistencies in the adoption of the DP coils and minimized the resultant field inhomogeneity. This is the first fabricated NMR applicable magnet with the use of Bi-2223 tapes and DP winding, providing practical techniques for building NMR magnets at ultra-high field strength. The magnet provides a useful tool for material science research in a synergetic extreme condition facility (SECF) in China, as well as providing a reference for future higher field strength NMR magnet development.

In order to establish a better relationship between brain functions and cellular environments, high spatial resolution is required when applying the techniques in functional magnetic resonance imaging (fMRI), diffusion-based tractography and susceptibility-weighted MRI to examine the characteristics of microstructures in brain tissue [14]. In light of the correlation between field strength and signal-to-noise ratio, past research indicates that a small voxel size of ∼100 μm can be achieved at an ultra-high field strength of 21.1 T with a significantly higher signal-to-noise ratio than the 9.4 T commonly used for preclinical research using small animals [15]. Therefore, an application of this highlighted study will be to provide peripheral components for the ultra-high field magnet and integrate it into an MRI system. With the combination of RF coils with high-density channels for imaging acceleration [16] and advanced magnetic shim techniques using integrated coils [17], the application areas of this type of magnet will be substantially broadened not only in condensed matter physics but also in the medical imaging domain in order to understand brain functions in preclinical research [18].

## Supplementary Material

nwae165_Supplemental_File
