# Supplementary material for: Fabrication of a 25.2 T NMR magnet for an extreme condition user facility in China
Source: Natl Sci Rev. 2024 May 10;11(7):nwae165. doi: 10.1093/nsr/nwae165 (PMC11177881; doi:10.1093/nsr/nwae165)
Supplement: nwae165_Supplemental_File [file nwae165_supplemental_file.docx]

Supplemental Table 1: The parameters of notch DP coils for the inner HTS magnet

|  |  |  |  |  |
| --- | --- | --- | --- | --- |
| Inner magnet | | | | |
| Coil no. | Turns | Internal diameter (mm) | External diameter (mm) | Thickness (mm) |
| N-I15 | 112.63 | 59.946 | 129.280 | 9.64 |
|  | 112.63 | 59.946 | 129.280 |  |
| N-I16 | 111.65 | 61.354 | 129.280 | 9.64 |
|  | 111.65 | 61.354 | 129.280 |  |
| N-I17 | 111.42 | 61.868 | 129.280 | 9.64 |
|  | 111.42 | 61.868 | 129.280 |  |
| N-I18 | 112.35 | 62.520 | 129.280 | 9.64 |
|  | 112.35 | 62.520 | 129.280 |  |
| N-I19 | 112.12 | 62.590 | 129.280 | 9.64 |
|  | 112.12 | 62.590 | 129.280 |  |
| N-I20 | 111.76 | 62.043 | 129.280 | 9.64 |
|  | 111.76 | 62.043 | 129.280 |  |
| N-I21 | 111.49 | 61.451 | 129.280 | 9.64 |
|  | 111.49 | 61.451 | 129.280 |  |
| N-I22 | 111.94 | 60.797 | 129.280 | 9.64 |
|  | 111.94 | 60.797 | 129.280 |  |
|  |  |  |  |  |
